# Supplementary material for: Dynalign II: common secondary structure prediction for RNA homologs with domain insertions
Source: Nucleic Acids Res. 2014 Nov 21;42(22):13939–48. doi: 10.1093/nar/gku1172 (PMC4267632; doi:10.1093/nar/gku1172)
Supplement: SUPPLEMENTARY DATA [file supp_gku1172_nar-02021-z-2014-File012.zip › manual/GUI/html/Tips_and_Techniques.html]

RNAstructure GUI Help -- Tips and Techniques


|  |  |  |
| --- | --- | --- |
|  | RNAstructure GUI Help Tips and Techniques | - Contents - Index |
| **Changing Temperature of Calculations**  To change the temperature at which calculations occur, choose the "Temperature" menu item. (This menu item only appears after a structure prediction module has been chosen.) A dialog will pop up showing the current temperature of calculations. This can be edited by changing the value in the text box and clicking "OK." If "Cancel" is selected, or an invalid temperature is entered, the temperature is not changed. Temperature changes should be used with caution. The enthalpy parameters for predicting free energy changes at temperatures other than 37 degrees C are prone to significant errors outside the range of about 20 to 50 degrees C. Note that the change in temperature applies only to a single calcution. Subsequent calculations will be performed at the default of 37 degrees C.   ---  **Changing the Maximum Pairing Distance**  A maximum pairing distance can be specified by choosing "Force -> Maximum Pairing Distance" and clicking "Yes" for "Limit Distance Between Paired Bases," then entering the preferred distance. (This menu item only appears after a structure prediction module has been chosen.) Nucleotides farther separated in sequence than the specified number will not be allowed to pair. The maximum distance constraint is not saved with other constraints.   ---  **Changing the Maximum Internal/Bulge Loop Size**  The maximum size of bulge or internal loops can be by choosing "Maximum Loop -> Set Maximum Loop Size" on the menu. (This menu item only appears after a structure prediction module has been chosen.) The default is 30, and this is sufficient for structure predictions.   ---  **Outputting Images**  RNAstructure can output images from the "Draw" and "Dot Plot" modules as Postscript. Select one of the associated options to export these images.   ---  **Using Folding and Alignment Constraints**  *Reviewing and Resetting Constraints*  To review current selections, choose "Force -> Show Current Constraints." To reset selections, select "Force -> Reset Current Constraints." (This menu item only appears after a structure prediction module has been chosen.)  Note that neither of these options include SHAPE constraints.   *Saving and Restoring Secondary Structure Prediction Constraints*  Folding constraints can be saved for future use or restored from previous saves using the "Force -> Save Constraints" and "Force -> Restore Constraints" menu options, respectively. (This menu item only appears after a structure prediction module has been chosen.) Constraint files are saved using the constraint file format. (See "Constraint File Format.")  As above, neither of these options include SHAPE constraints.   *Forcing a Base Pair*  Helixes can be forced to occur in secondary structure prediction. After selecting the sequence to fold, choose the menu option "Force -> Base Pair." (This menu item only appears after a structure prediction module has been chosen.) Under "Base 1," indicate the number of the 5' most base to be paired. Under "Base 2," indicate the 3' most base to be paired. Under "Helix Length," enter the number of base pairs long the forced helix must be.  For example, if 17 was indicated for the first base in pair, and 83 for the second base in pair, with a helix length of 5, a helix would occur with these base pairs: 17-83, 18-82, 19-81, 20-80, and 21-79.  Note that it is important that the nucleotides forced to pair can form a canonical pair (A-U, G-C, or G-U). If not, no structure will be predicted. Also, RNAstructure does not allow base pairs at positions that cannot stack, i.e. be directly adjacent to, another canonical pair. If a pair is forced between nucleotides that cannot stack on an adjacent pair, then no structure will be predicted.   *Forbidding a Base Pair*  Helixes can be specified as forbidden in secondary structure prediction. After selecting the sequence to fold, choose the menu option "Force -> Prohibit Base Pairs." (This menu item only appears after a structure prediction module has been chosen.) Under "Base 1," indicate the number of the 5' most base of the forbidden helix. Under "Base 2," indicate the 3' most base of the forbidden helix. Under "Helix Length," enter the length of the helix that is to be forbidden.  For example, if 17 was indicated for the first base in pair, and 83 for the second base in pair, with a helix length of 5, the following base pairs would be prohibited: 17-83, 18-82, 19-81, 20-80, and 21-79.   *Forcing Single Stranded Bases*  Bases can be selected that should not pair in the secondary structure prediction. This can be done in one of two ways:   1. A base can appear as lowercase in the sequence file (see Sequence File Format). 2. The "Force -> Single Stranded" menu option can also be chosen. (This menu item only appears after a structure prediction module has been chosen.) Into the "Base Number" box, enter the number indicating the position from the 5' end of the sequence of a base that should be single-stranded. Click "OK" and the dialog will reset to take another forced single-stranded nucleotide. Click "OK and Close" after all single-stranded nucleotides have been entered.    *Forcing Double Stranded Bases*  Bases can be selected that must be base paired. This is done with the "Force -> Double Stranded" menu. (This menu item only appears after a structure prediction module has been chosen.) It is analogous to forcing a base to be single stranded.   *Specifying Nucleotides Accessible to Chemical Modification*  Nucleotides can be selected that are accessible to chemical modification. These nucleotides can be located in a loop, at the end of a helix, in a GU pair, or adjacent to a GU pair.  Select the "Force -> Chemical Modification" menu option. (This menu item only appears after a structure prediction module has been chosen.) Into the "Base Number" box, enter the number indicating the position from the 5' end of a modified nucleotide. Click "OK" and the dialog will reset to take another nucleotide. Click "OK and Close" after all chemically modified nucleotides have been entered.   *Specifying FMN Cleavage (U's in GU pairs)*  Select the "Force -> FMN Cleavage" menu option. (This menu item only appears after a structure prediction module has been chosen.) Into the "Base Number" box, enter the number indicating the position from the 5' end of a U in a GU pair. Click "OK" and the dialog will reset to take another nucleotide. Click "OK and Close" after all nucleotides have been entered.   *A Note About Adding Multiple Folding Constraints Files*  If a constraints file is specified after constraints have already been appplied, either by a constraints file or manual input, all former constraints are erased in favor of the data in the new constraints file  *Specifying SHAPE Mapping Data*  See "SHAPE File Format" for details on the file type used for these constraints. Two methods exist to enter SHAPE data, as follows:   1. The preferred method uses a pseudo free energy change to influence the structure prediction. To use this method, choose the menu option "Force -> Read SHAPE Reactivity -- Pseudo-Energy Constraints." (This menu item only appears after a structure prediction module has been chosen.) First, choose a data file by clicking the "SHAPE Data File" button. Then, choose values for the "Slope" and "Intercept" boxes (defaults are provided). Click "OK" when finished. 2. The less preferred method is a hard cutoff method. To use this method, choose the menu option "Force -> Read SHAPE Reactivity -- Hard Constraints." (This menu item only appears after a structure prediction module has been chosen.) First, choose a data file as specified in the previous method. Then, choose values for the "Threshold for Force Single Stranded" and "Threshold for Chemical Modification" boxes (defaults are provided). Nucleotides with SHAPE reactivity above "Threshold for Force Single Stranded" will be forced single stranded. Nucleotides with SHAPE reactivity between "Threshold for Chemical Modification" and "Threshold for Force Single Stranded" will be treated as though they are nucleotides accessible to chemical modification. | | |
| Visit The Mathews Lab RNAstructure Page for updates and latest information. | | |
